# Kidney Disease in Diabetic Patients: From Pathophysiology to Pharmacological Aspects with a Focus on Therapeutic Inertia

**DOI:** 10.3390/ijms22094824

**Published:** 2021-05-01

**Authors:** Guido Gembillo, Ylenia Ingrasciotta, Salvatore Crisafulli, Nicoletta Luxi, Rossella Siligato, Domenico Santoro, Gianluca Trifirò

**Affiliations:** 1 Unit of Nephrology and Dialysis, Department of Clinical and Experimental Medicine, University of Messina, 98125 Messina, Italy; guidogembillo@live.it (G.G.); rossellasiligato@gmail.com (R.S.); 2 Department of Biomedical and Dental Sciences and Morpho-Functional Imaging, University of Messina, 98125 Messina, Italy; ylenia.ingrasciotta@unime.it (Y.I.); crisafulli.salvatore@unime.it (S.C.); 3 Department of Diagnostics and Public Health, University of Verona, 37100 Verona, Italy; nicoletta.luxi@unime.it (N.L.); gianluca.trifiro@univr.it (G.T.)

**Keywords:** diabetic kidney disease, diabetes, therapeutic inertia, end-stage renal disease, diabetic nephropathy, antidiabetic drugs

## Abstract

Diabetes mellitus represents a growing concern, both for public economy and global health. In fact, it can lead to insidious macrovascular and microvascular complications, impacting negatively on patients’ quality of life. Diabetic patients often present diabetic kidney disease (DKD), a burdensome complication that can be silent for years. The average time of onset of kidney impairment in diabetic patients is about 7–10 years. The clinical impact of DKD is dangerous not only for the risk of progression to end-stage renal disease and therefore to renal replacement therapies, but also because of the associated increase in cardiovascular events. An early recognition of risk factors for DKD progression can be decisive in decreasing morbidity and mortality. DKD presents patient-related, clinician-related, and system-related issues. All these problems are translated into therapeutic inertia, which is defined as the failure to initiate or intensify therapy on time according to evidence-based clinical guidelines. Therapeutic inertia can be resolved by a multidisciplinary pool of healthcare experts. The timing of intensification of treatment, the transition to the best therapy, and dietetic strategies must be provided by a multidisciplinary team, driving the patients to the glycemic target and delaying or overcoming DKD-related complications. A timely nephrological evaluation can also guarantee adequate information to choose the right renal replacement therapy at the right time in case of renal impairment progression.

## 1. Introduction

Diabetes mellitus (DM) is a metabolic disease characterized by chronic hyperglycemia as a consequence of defects in insulin action, secretion, or both. DM currently affects more than 463 million people worldwide (9.3% of adults aged 20–79 years), and the number of patients with DM is estimated to rise up to 578 million by 2030, and 700 million by 2045 [1]. The World Health Organization (WHO) reported that DM is the leading cause of kidney failure globally [2]. Specifically, diabetic kidney disease (DKD), which is defined as elevated urine albumin excretion or reduced glomerular filtration rate (GFR) or both, is a serious complication that occurs in up to 40% of all diabetic patients [3].

The clinical and socio-economic impact of DKD is burdensome not only because of the risk of progression to end-stage renal disease (ESRD) and therefore to renal replacement therapies, but also because of the associated increase in cardiovascular (CV) risk [4,5]. A strict control of blood glucose is essential in DKD. Although many antidiabetic agents are currently available, the treatment of diabetes in DKD is challenging. Many antidiabetic drugs are contraindicated in advanced CKD, and others require dose adjustments due to an increased risk of drug toxicity as a result of reduced renal excretion [6,7].

Based on established vascular complications and comorbidities, it is necessary to identify treatment targets, and many trials [8,9,10] suggest that delayed treatment intensification can lead to irreversible diabetes-related complications [11]. This was confirmed by a retrospective cohort study showing that a 1-year delay in treatment intensification in uncontrolled patients significantly increased the risk of myocardial infarction, heart failure, stroke, and a composite endpoint of CV events [12].

The failure to establish appropriate targets and escalate treatment to achieve treatment goals timely is termed “therapeutic inertia”. A broader concept of “clinical inertia” includes the delay of the escalation or deintensification of therapy and issues such as failure to screen, make appropriate referrals, and manage risk factors and complications [13]. Moreover, Phillips et al. described the concept of clinical inertia as “failure of health care providers to initiate or intensify therapy when indicated” [14]. Clinical inertia could be responsible for substantial preventable complications of diabetes, including DKD, with the associated excess in direct and indirect health care costs. In particular, it accounts for a significant proportion of failure to achieve targets in the diabetes management and contributes to up to 200,000 adverse diabetes-related outcomes per year in North America [15].

This narrative review aims to provide an overview of the pathophysiology and pharmacological management of DKD, highlighting evidence on therapeutic inertia from real-world settings and discussing barriers and potential strategies to optimize DKD management.

## 2. DKD Epidemiology and Risk Factors

The number of patients with CKD is progressively increasing, and DKD plays a central role in the progression to ESRD. KDIGO guidelines define CKD as persistent abnormalities of kidney structure or function, or both, for more than 3 months. CKD is further classified based on an elevated urine albumin/creatinine ratio (UACR) (≥30 mg/g [3 mg/mmol]) and a reduced estimated GFR (GFR < 60 mL/min per 1.73 m^2^) [16].

Traditionally, DKD can be identified by the presence of abnormal UACR [17] and defined by the triad of albuminuria, hypertension, and decline of renal function in diabetic patients [18]. However, the UK National Diabetes Audit noticed that 54.5% of type 1 DM (T1DM) patients and 63.7% of type 2 DM (T2DM) patients registered a reduced GFR with a normal UACR. This evidence highlighted that many DM patients have DKD without the presence of albuminuria [19]. Novel biomarkers and high-dimensional panels with high sensibility and specificity are still under study [20].

Due to the differences in economic viability and governmental infrastructures between countries, there is a marked racial/ethnic difference besides the international difference in the epidemiology of diabetic nephropathy [21]. DKD is more frequent in African-Americans, Asian-Americans, and Native Americans than in non-Hispanic whites with T2DM [22]. Progressive kidney disease is more frequent in Caucasian patients with T1DM than in those with T2DM, although its overall prevalence in the diabetic population is higher in patients with T2DM because this type of DM is more prevalent [23].

The Institute for Alternative Futures (IAF) calculated that the number of DM patients in the United States would increase by 54%, between 2015 and 2030, with a rise of diabetes-related mortality of 38% [24,25,26]. DKD also represents the leading cause of CKD worldwide, developing approximately in 40% of diabetic patients [27]. A proper strategy to improve DM and DKD prevention and treatment is of pivotal importance to contrast this pandemic emergency.

An analysis of the United States Renal Data System reported that DKD-related deaths has been increasing, while other causes of CKD have been relatively stable [28]. Gender-related outcomes in DKD are still under debate and researchers are still exploring the different causes of this gender variability [29]. The Global Burden of Diseases 2017 Study results demonstrated a DKD prevalence equal to 15.5/1000 in men and 16.5/1000 in women. In this gender-related analysis, Oceania was the world region with the highest prevalence of DKD, while the lowest prevalence was in Western Europe for both sexes [30].

T2DM also presents a heritable polygenic component with specific susceptibility loci [31,32,33], which confers to about 40% of first-degree relatives the risk of developing the same affection, as compared to a 6% incidence in the general population [34]. DKD is also influenced by epigenetic mechanisms involving chromatin histone modulations, DNA methylation, and the influence of non-coding RNA action [35].

Both genetic and environmental variabilities represent risk factors of disease progression. Besides the non-modifiable risk factors, such as family history, genetics, gender, age at diagnosis, and DM duration, lifestyle can be improved promoting healthy habits. It is important to maintain a proper glycemic control, blood pressure, avoid or quit smoking, reduce alcohol consumption, practice physical activity, follow a balanced diet and maintain a healthy lipidic profile [36].

Several epidemiological studies indicated obesity as one of the main risk factors for insulin resistance development and T2DM progression [37]. According to the WHO, almost 90% of DM subjects develop T2DM contextually to a body overweight status [38].

It is of paramount importance to guarantee a structured education for patients and health care professionals to raise awareness to the role of DM and DKD prevention. Self-management knowledge should be used as an adjunct therapeutic option, especially in high-risk patients.

## 3. Pathophysiology of DKD

The pathophysiology of DKD is multifactorial and characterized by a critical metabolic impairment; the upstream influence of hyperglycemia leads to a dysregulated intracellular metabolism, inflammatory lesions, increased apoptosis processes and tissue fibrosis [39]. At the basis of DKD injury there are three crucial steps: (1) glomerular hypertrophy leading to hyperfiltration. Glomerular hyperfiltration is present in up to 75% of T1DM patients and up to 40% of patients with T2DM and is a typical feature of early DKD manifestations [27]; (2) glomerular and tubulointerstitial inflammation, related to chemokines, cytokines, and profibrotic factors activation; (3) dysregulated cellular apoptosis and changes in the extracellular matrix. These mechanisms lead to glomerular basement membrane thickening, podocyte depletion, mesangial matrix expansion, and tubular damage. All these factors may contribute to the progression of DKD, resulting in vascular remodeling, endothelial dysfunction, glomerulosclerosis, and tubulointerstitial fibrosis [40,41,42].

Different intracellular pathways demonstrated a driving role in the DKD process, stimulated by hyperglycemia. High blood glucose stimulates protein kinase C beta type (PKC-beta) and protein kinase C delta type (PKC-delta) activation in the renal cortex. This mechanism triggers the nuclear factor kappa-light-chain-enhancer of activated B cells (NF-κB) and the release of both interleukin (IL)-6 and the tumor necrosis factor (TNF)-α by endothelial and mesangial cells [43,44]. The advanced glycation end-products species (AGEs) pathway not only alters the reactive oxygen homeostasis in a pro-oxidant way [45,46] but also contributes to the ultrastructural changes of the mesangial matrix, with a preferential localization to nodular lesions of DKD patients [47]. PKC and AGEs pathways have one trigger factor in common, the accumulation of glyceraldehyde-3-P: this enzyme-substrate activates the AGEs pathway stimulating the production of methylglyoxal from non-enzymatic dephosphorylation of the triose phosphates and triggers the PKC pathway, stimulating the synthesis of diacylglycerol [48]. Glyceraldehyde-3-P promotes the production of glycolytic metabolites upstream, triggering more pro-oxidative pathways, such as hexosamine [49] and polyol [50] pathways.

In addition to PKC and AGEs-guided mechanisms, more intracellular pathways seem to be implicated in the DKD insult. NF-κB, inducible nitric oxide synthase, JAK/STAT, and transforming growth factor-beta1/SMAD pathways are all leading to the production of proinflammatory molecules inducing extracellular matrix deposition and the differentiation/proliferation of myofibroblast in DKD patients [51,52,53,54].

## 4. Management of Diabetes Mellitus in the Transition from DKD to ESRD

DKD is an insidious complication of diabetes, often silent for years. The average time of onset of kidney impairment in diabetic patients is about 7–10 years [55]. A timely recognition of the risk factors for DKD progression can be crucial in decreasing morbidity and mortality in diabetic patients.

Several wake-up calls should alarm diabetic patients regarding their kidneys’ health, and patients should be referred to a nephrologist earlier if they present rapid renal reduction, resistant hypertension, hyperkalemia, UACR exceeding 300 mg/g, or other urinary abnormalities [56].

Some real-world experiences, such as that of Martínez-Castelao et al., report a late referral of patients to specialist nephrology clinics when the kidney mass is already reduced by 70% [57]. An on-time nephrologist’s evaluation could lower the rates of undertreatment [58] and reduce ESRD incidence and related mortality [59].

A proper remodeling of lowering glucose therapy is one of the main points that should be evaluated in the evolution from DKD to ESRD. Diabetic patients with ESRD present high levels of blood urea nitrogen, leading to carbamylated hemoglobin production; these molecules are not distinguishable from glycosylated hemoglobin by electrophoresis, causing incorrect elevated levels of hemoglobin A1C [60]. Moreover, the reduced lifespan of red blood cells, iron deficiency, and erythropoietin-stimulating agents can lead to an undervaluation of glucose control [61].

Most oral diabetes drugs are contraindicated in ESRD and the pharmacological therapy should be balanced to avoid over- and undertreatment. Individuals with impaired renal function have a higher risk of lactic acidosis related to metformin use, which should be used cautiously in patients with a progressive decrement of renal function [62]. Moreover, ESRD patients present impaired gluconeogenesis control and reduced insulin clearance. Patients with GFR <20 mL/min present a decrease in the hepatic metabolism of insulin, a condition worsened by the action of uremic toxins on the liver, thus requiring a reduced exogenous insulin dose according to their renal impairment, in order to avoid hypoglycemia [63,64].

The American Diabetes Association suggests that diabetic patients should be evaluated for renal replacement therapy when the GFR falls below 30 mL/min/1.73 m^2^ [65].

In CKD patients, a healthy transition program leads to a lower number of hospitalizations and dialysis emergency start, as well as to lower catheter use with a higher use of arterial-venous fistulas [66]. These models should be used and improved in DKD patients, where a further pharmacological check and the remodulation of diabetic medications should be performed according to the patient’s renal function.

For DKD patients, the transitional ambulatory can represent an opportunity to be evaluated also for non-pharmacological treatments. Renal pre-emptive transplantation or combined pancreas-renal transplantation can represent a suitable option for selected subjects, especially for T1DM patients. Despite the significant improvement in DKD treatment in the last decades, these patients remain at higher risk of ESRD development and mortality; a pre-emptive transplant can strongly improve their quality of life and life expectancy [67]. The study of Piccoli et al. [68] indicated that at referral to the nephrologist, over 50% of T1DM patients might have indications for pancreas-kidney or pancreas transplantation. A multidisciplinary evaluation on time can represent a keystone in the implementation of DKD patient care. A potential alternative to dialysis can encourage diabetic patients with renal impairment. This awareness and faith in the future can lead the patients to be more compliant with the therapy and to trust the physicians.

## 5. Pharmacological Management of DKD—New Insights and Old Confirmations

### 5.1. RAS Blockade

DKD is a crucial harm in patients affected by DM because it represents a risk of CKD progression up to ESRD and increased CV morbidity and mortality. DKD treatment addresses both problems with first-choice drugs represented by renin-angiotensin system (RAS) blockade, including either angiotensin-converting enzyme inhibitors (ACEi) or angiotensin II receptor blockers (ARB). These drugs played a pivotal role in reducing albuminuria and slowing GFR losses in several clinical trials, such as the Collaborative study (captopril) [69], RENAAL (losartan) [70], and the IRMA and IDNT studies (irbesartan) [71,72]. A Cochrane systematic review, published in 2006, concluded that ACEi or ARB versus placebo were associated with a statistically significant reduction of ESRD risk (relative risk (RR): 0.60; 95%CI: 0.39–0.93 and RR: 0.78; 95% confidence interval (CI): 0.67–0.91, respectively), macroalbuminuria (RR: 0.45; 95%CI: 0.29–0.69 and RR: 0.49; 95%CI: 0.32–0.75, respectively), as well as an increased regression of micro- to normo-albuminuria (RR: 3.06; 95%CI: 1.76–5.35 and RR: 1.42; 95%CI: 1.05–1.93) [73]. According to the latest KDIGO guidelines, RAS blockade is recommended at the maximum tolerated dose in all the patients affected by both hypertension and albuminuria. Moreover, considering the ACEi and ARB anti-proteinuric effect, it should be evaluated even in normotensive subjects [74].

Particular attention should be paid to transient changes in the serum levels of potassium and creatinine after RAS blockade introduction. The KDIGO guidelines advise nephrologists to lower the RAS blockade dose only in symptomatic hypotension, uncontrolled hyperkalemia, or >30% rise in serum creatinine levels. Hyperkalemia is a common effect of high-dose RAS blockade; to avoid losing potential beneficial effects on proteinuria, it is preferable to adopt cation-exchange resins such as sodium polystyrene sulfonate or calcium polystyrene sulfonate. This can represent a valid strategy to maintain potassium levels in a normal range rather than reducing doses or suspending ACEi or ARB as a first step.

Nephrologists should warn patients that the rise of up to 30% in serum creatinine level 4 weeks after ACEi or ARB treatment start may be reversible after their discontinuation, especially in patients affected by moderate-severe CKD stages. A different consideration regards patients in whom the association of RAS blockade with either aggressive diuretic therapy or non-steroidal anti-inflammatory drugs may increase the risk of developing acute kidney injury (AKI). Renal artery stenosis might also cause a sudden rise of serum creatinine after RAS blockade administration, especially in smokers or patients affected by atherosclerotic cardiovascular disease (ASCVD), and has to be ruled out in case of AKI.

A dual blockade with ACEi/ARB or their association with either mineralocorticoid receptor antagonists (MRA) or a renin inhibitor is also discouraged. Finerenone, a new non-steroidal MRA with higher mineralocorticoid receptor selectivity, was shown to reduce albuminuria in a dose-dependent manner in a cohort of DKD patients treated with RAS blockade, with a small incidence of hyperkalemia (3%) [75].

FIDELIO-DKD and FIGARO-DKD are currently under study in phase 3 trials, involving DKD patients with GFR 25–60 mL/min/m^2^ and a baseline ACEI/ARB therapy, to investigate their protective effect on renal function and on CV events [76,77,78]. FIDELIO-DKD showed a significant reduction of risk of kidney failure, a sustained decrease of at least 40% in the GFR from baseline, or death from renal causes (hazard ratio (HR): 0.82; 95%CI 0.73–0.93; *p* = 0.001), and a lower risk of death from CV causes, non-fatal myocardial infarction or stroke, or hospitalization due to heart failure (HR 0.86, 95%CI 0.75–0.99; *p* = 0.03).

Particular attention has to be paid in women in fertile age because of the known teratogen effects of ACEi, which should be discontinued as soon as patients become pregnant.

### 5.2. Antidiabetic Drugs

Due to the reduced renal excretion, many antidiabetic drugs (substantially excreted via the kidney) are contraindicated or require dose adjustments in DKD patients to prevent hypoglycemia [74,79,80] (Table 1). The latest KDIGO guidelines recommend the use of metformin together with Sodium-glucose co-transporter-2 inhibitors (SGLT2i) as the first-line therapy due to their cardioprotective effects and preventive effects on CKD progression in patients with GFR ≥30 mL/min/1.73 m^2^ [74]. Metformin has been shown to be safe and effective in glycemic control in patients with T2DM, but it is contraindicated if GFR <30 mL/min/1.73 m^2^; SGLT2i, on the other hand, have low hypoglycemic effect in patients with impaired renal function, and therefore their use should be restricted in such patients [74,79].

Considering its low risk of inducing hypoglycemia even in mildly impaired renal function, as well as its wide availability and low cost, metformin is the first-line therapy in most T2DM patients. The United Kingdom Prospective Diabetes Study first demonstrated its superiority over sulfonylureas or insulin in reducing CV risk in T2DM obese patients, as confirmed in a systematic review conducted by Marunthur et al., who detected a reduction in CV mortality with an RR of 0.6–0.7 from RCTs in favor of metformin compared with sulfonylureas [81].

No RCTs are currently available evaluating metformin CV protection in CKD patients. A systematic review of six observational studies conducted on patients affected by moderate to severe CKD showed a 22% lower risk of all-cause mortality in the metformin cohorts than in other antihyperglycemic drugs (HR 0.78; 95% CI 0.63 to 0.96; Q = 29.7 [*p* < 0.001], I2 = 79.8%), which was more evident in subjects with GFR 45–60 mL/min/1.73 m^2^ than with 30–45 mL/min/1.73 m^2^. However, this review offers a low level of evidence [82]. Moreover, kidney transplant recipients should be given metformin following the KDIGO guidelines’ indications on its suspension only for GFR <30 mL/min/1.73 m^2^, because no evidence of adverse effects have been reported on allografts in mild CKD up to now.

SGLT2i are drawing the attention of the medical community because of their CV and renal protective effect more than their modest antihyperglycemic potential, estimated in a meta-analysis by Vasilakou et al. as a mean HbA1c difference in treated patients vs. placebo of −0.66% (95% CI, −0.73% to −0.58%); vs. other drugs, −0.06% (CI, −0.18% to 0.05%). SGLT2i lower glucose blood levels by inhibiting the renal tubular reabsorption of glucose, causing osmotic diuresis. Moreover, they appear to reduce the intraglomerular pressure, thus correcting hyperfiltration, which is at the basis of DKD development and progression [83]. On the other hand, glycosuria may increase the risk of a genito-urinary tract infection, thus making these drugs unsuitable for treating T2DM in transplanted patients [74].

The first RCTs on T2DM also including patients with GFR ≥30 mL/min/1.73 m^2^ (Empagliflozin Cardiovascular Outcome Event Trial in Type 2 Diabetes Mellitus Patients–Removing Excess Glucose (EMPAREG), CANagliflozin cardioVascular Assessment Study (CANVAS), and Canagliflozin and Renal Endpoints in Diabetes with Established Nephropathy Clinical Evaluation (CREDENCE)) or ≥60 mL/min/1.73 m^2^ (Dapagliflozin Effect on CardiovascuLAR Events (DECLARE-TIMI 58)) [83,84,85,86] reported a reduced rate of CV or kidney adverse outcomes described as the doubling of serum creatinine, ESRD or renal death in all patients treated with SGLT2i. Due to the relatively small number of DKD patients in each RCT, Zelniker et al. performed a meta-analysis of EMPA-REG, CANVAS, and DECLARE-TIMI 58 data to stratify outcomes among individuals with and without CKD reaching statistical significance [84]. In patients with a GFR of 30 to <60 mL/min per 1.73 m^2^, SGLT2i reduced the risk of major adverse CV events (HR 0.82; 95% CI: 0.70–0.95). Interestingly, CKD progression reduction (HR 0.55, 95% CI 0.48–0.64, *p* < 0.0001) and hospitalization for heart failure (HR 0.60; 95% CI: 0.47–0.77) varied according to the CKD stage at enrollment, with less beneficial effects in subjects with more severe kidney impairment.

Overall, metformin and SGLT2i are contraindicated in patients with GFR <30 mL/min/1.73 m^2^ [74,79,80]. In these cases, other antidiabetic drugs are needed for glycemic control, considering the patient’s conditions.

Generally, glucagon-like peptide-1 receptor agonists (GLP-1 RA) are recommended in these situations due to their CV and benefits on albuminuria [74]. GLP-1 is an incretin hormone secreted by the intestine after a meal, in order to enhance the glucose-dependent release of insulin; it also decreases hunger stimulation, retards gastric emptying, and facilitates weight loss. As demonstrated by Kristensen et al. in a recent meta-analysis of the outcomes of seven RCTs involving GLP-1 RA (ELIXA, LEADER, SUSTAIN-6, EXSCEL, HARMONY, REWIND, and PIONEER 6) vs. placebo, the GLP-1 RA treatment reduces the risk of a composite kidney outcome (HR: 0.83; 95% CI: 0.78–0.89) mainly regarding the development of new severely increased albuminuria and, to a lesser extent, a rise in serum creatinine or GFR loss, progression to ESRD, and renal death (HR: 0.87; 95% CI: 0.73–1.03, lacking statistical significance) [85].

Dipeptidyl peptidase-4 inhibitors (DPP-4i) prolong the activity of GLP-1 by inhibiting its catabolism and should be used with adjusted dosage regimens in DKD patients. They demonstrated a favorable safety profile and a very low risk of hypoglycemia [74]. DPP-4i may improve two major risk factors for DKD, such as hyperglycemia and albuminuria, but RCTs are inconclusive about hard kidney outcomes [86].

Sulfonylureas induce insulin release by preventing potassium from exiting in pancreas beta-cells. The consequent cell depolarization causes the opening of calcium channels leading to an increased insulin release. First-generation sulfonylureas should be avoided in DKD patients. Concerning second-generation sulfonylureas, glipizide and gliclazide do not require dose adjustment, while glimepiride does; glyburide is contraindicated in DKD patients [74,79,80].

The use of acarbose, an alpha-glucosidase inhibitor, should be avoided in patients with DKD [79,80].

Thiazolidinediones are ligands of peroxisome proliferator-activated receptors γ (PPAR γ) and activate glycemic control and lipid homeostasis. They are not contraindicated in patients with DKD [79,80] because of their protective effect in DKD, preventing or delaying its progression [87,88,89]; however, due to their adverse effects (e.g., fluid retention), caution should be exercised in case of advanced renal dysfunction associated with an ineffective diuresis [80].

Due to its short duration of action and transformation into inactive metabolites excreted in the feces, repaglinide can be used in patients with DKD, without dose adjustment [79,80].

Concerning insulin, although there are no guidelines on what type of insulin should be used or avoided, insulin treatment is considered safe in patients with DKD [74,80]. However, for this drug class, a dose adjustment based on each patient’s response could be considered [79,80].

### 5.3. Dyslipidemia Management

Elevated levels of triglycerides and low-density lipoprotein—cholesterol (LDL-c) are associated with an increased CV risk and the progression of CKD in patients with DKD. Thus, an evaluation of the lipid profile is indicated, and an appropriate pharmacological approach in patients with DKD is needed. Lipid-lowering therapy with statins was proven to have a protective effect on renal function by improving albuminuria and the estimated GFR [90]. However, since high doses of statins may be toxic in patients with GFR <60 mL/min/1.73 m^2^, a dose adjustment is required [90] based on each patient’s GFR [90] (Table 2). On the other hand, KDIGO guidelines suggest that statin treatment should not be started in DKD patients on dialysis [91].

### 5.4. Antiplatelet Therapy

Antiplatelet agents are widely used in the secondary prevention of CV disease. DKD patients are at higher risk of thrombo-embolic events. However, these patients are also at high risk of bleeding. Therefore, evidence suggests that the use of antiplatelet agents in a multi-drug approach is effective in reducing CV risk. However, antiplatelet therapy as a primary prevention is to be avoided in patients with DKD [92].

## 6. Critical Issues on DKD Management: Evidence from Real-World Settings

Glycemic control in DKD patients is strongly recommended not only for cardiovascular prevention, but also to prevent DKD progression [93]. Glycemic management in patients affected by DKD is challenging due to several factors, such as therapeutic inertia, monitoring difficulties, and the complexity regarding the use of the available treatments [94]. Indeed, one of the main issues in glycemic control in DKD patients is that the risk of hypoglycemia increases with a decreasing GFR, mainly because of the altered pharmacodynamic and pharmacokinetic profiles of antidiabetic drugs and the reduced kidney mass [95].

Several real-world studies showed that renal impairment is often not sufficiently taken into account for adjusting the dose of antidiabetic drugs that are contraindicated in DKD [7,96,97,98]. In 2011, a retrospective observational study conducted by Meyers et al. reported that metformin and sitagliptin were frequently used at inappropriate doses in patients with renal impairment [96]. The OREDIA study, a French multicentric, cross-sectional observational study conducted between 2012 and 2013, showed that around one-third of 2472 patients with T2DM and CKD were still treated with metformin without dose adjustment and that antidiabetic drugs with a high hypoglycemia risk were still heavily prescribed in this population [97]. Similar scenarios were observed in three more recent observational studies published in 2016 [7,99] and 2018 [98]. Specifically, Trifirò et al. found that, in a general population of Southern Italy, the treatment of DM among CKD patients changed only marginally after the diagnosis of CKD, with a slight reduction of metformin use and an increase in the use of insulin and repaglinide [7]. Results from a French prospective observational study describing the prescribing practice patterns according to GFR show a considerable number of inappropriate prescriptions of oral antidiabetic drugs among T2DM patients with CKD, mostly concerning metformin (30% of the whole study cohort) and sitagliptin (17.9% of the whole study cohort) [99]. Issues in the management of T2DM patients with CKD were also documented by an observational study conducted by Min et al., in which a substantial proportion of DKD patients were treated with metformin and DPP-4i without dose adjustment [98].

A very recent population-based Italian study showed that among 336 patients starting the treatment with antidiabetic dugs different from metformin and for which information of the CKD stage was available, 137 (40.8%) had a diagnosis of severe renal impairment (e.g., CKD stage IV–V or dialysis) and were therefore not eligible for the treatment with metformin [100].

Along with glycemic control, the control of blood pressure and blood cholesterol levels is crucial to slow DKD progression and prevent its macrovascular and microvascular complications [101,102]. It has been demonstrated that maintaining blood pressure values below 140/85 mmHg is associated with a statistically significant reduction in the incidence of DKD in patients with hypertension and T2DM over a 4-year follow-up [103]. However, real-world evidence suggests that, despite the improvements in risk-factor control and diabetes management, glycemic, blood pressure, and LDL-cholesterol target levels are achieved only in a small proportion of T2DM patients, especially if concomitantly affected by CKD [101,104,105,106]. These gaps in diabetes care might be generally explained by several factors, such as the patients’ lack of motivation, the therapeutic inertia by the care providers, and, more generally, the logistical or financial barriers in the patients’ access to care [101]. In 2019, the ARETAEUS study results showed a delayed dyslipidemia treatment in a large cohort of T2DM patients, despite the presence of high LDL-cholesterol levels, either before or after the diagnosis of T2DM. Moreover, the antidiabetic treatment was not intensified when the glycemic targets were not reached [107]. The analysis of the indicators of therapeutic inertia using data from the Annals of the *Associazione Medici Diabetologi*, involving more than 300 diabetes centers throughout Italy, showed that between 2011 and 2018 the proportion of subjects with glycated hemoglobin >9% not treated with insulin had fallen from 40.5% to 28.2%, while there was no significant change in the ratio of subjects not treated with statins despite elevated LDL cholesterol levels (from 57.5% to 52.4%). Similarly, a considerable proportion of patients did not receive anti-hypertensive medication despite blood pressure values ≥140/90 mmHg (30.2% in 2011 vs. 26.2% in 2018); moreover, among subjects receiving anti-hypertensive medication, almost one out of two continued to have blood pressure values ≥140/90 mmHg (56.8% in 2011 vs. 48.5% in 2018) [108].

Due to their complex clinical conditions, DKD patients generally take many drugs to slow the progression of their renal disease, prevent specific complications, and manage comorbidities [109], thus leading to an increased risk of experiencing adverse drug reactions (ADRs) and drug-drug interactions. Moreover, the worsening of renal function is often caused by the use of nephrotoxic drugs, especially when used for a long period and at high dosages [110]. All these factors make appropriate drug prescribing more challenging in such a population of patients. It has been previously reported that between 15% and 67% of prescriptions in patients with renal impairment were inappropriate in terms of doses or concerned nephrotoxic drugs [111,112]. An Italian retrospective population-based study conducted on 2128 patients with CKD found that 49.8% and 45.2% of patients received at least one prescription of nephrotoxic drugs within one year before and after the first CKD diagnosis, respectively. Specifically, nonsteroidal anti-inflammatory drugs were the most prescribed nephrotoxic drugs to CKD patients, with nimesulide and diclofenac being most frequently used [113]. A recent cross-sectional analysis of the medication profiles of 556 patients diagnosed with CKD documented that 77% of them had at least one drug classified as renally-inappropriate, accounting for 31.3% of the drugs prescribed, and 9.25% were contraindicated drugs [114].

## 7. Factors Related to Therapeutic Inertia

Several factors may influence the need for the intensification of treatment, including ineffective diet and exercise initiatives, limited pharmacologic armamentarium, conservative management, adverse events, poor compliance, underlying physiopathology, limited resources, and suboptimal healthcare systems [115].

**Figure 1:**
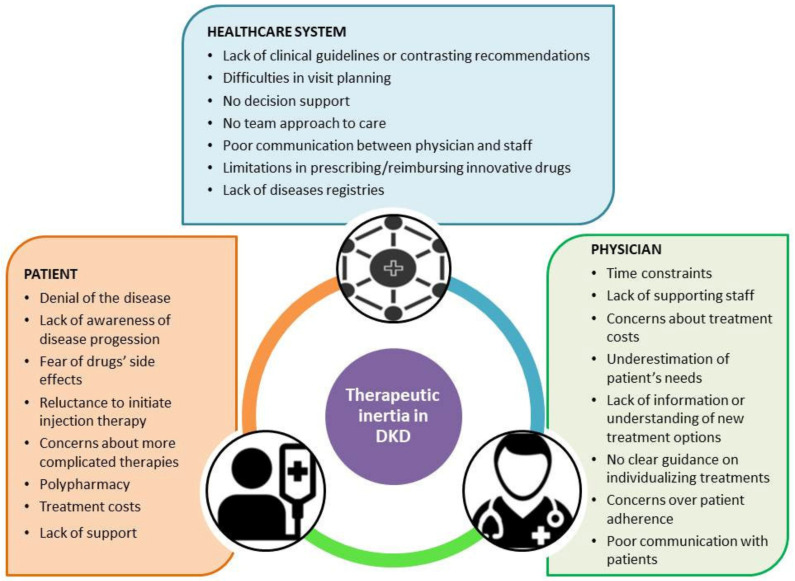
Factors related to therapeutic inertia.

Barriers to treatment intensification can be categorized into three levels (Figure 1):

(a) patient level: difficulty in changing lifestyle and taking the medication is common and is a significant contributor to the challenge of meeting glycemic targets. Moreover, the term “psychological insulin resistance” was used to describe patients’ refusal to start insulin therapy when recommended by a clinician. Depression is very common among people with DM, with reported rates as high as 17.8% compared with 9.8% in those without DM, and patients with depression are more likely to have concerns related to starting the treatment with insulin [105,116]. Elderly patients may struggle with vision impairment, limiting their ability to monitor glucose and use injectable medications [117]. The cost is also a concern for many patients and must be considered when choosing the therapy, particularly given the large difference between the cost of old versus new antidiabetic drugs [118]. Specifically, patients with lower incomes and higher out-of-pocket costs are likely to forego or be less likely to take antidiabetic drugs. Other patient-related factors (e.g., fear of hypoglycemia, weight gain or beliefs that insulin therapy is not efficacious [119], or fear that their quality of life will drop considerably [119,120]) can contribute to therapeutic inertia.

(b) clinician level: several provider-related factors can lead to therapeutic inertia: overestimating the quality of care, lack of materials, lack of time available to communicate with the patient, and training to escalate care to meet the recommended targets appropriately. The lack of knowledge and resources also delays treatment intensification, particularly insulin initiation. Studies comparing General Practitioners (GPs) to specialists have shown that the latter are more likely to initiate insulin and GLP-1 RA earlier in the course of therapy than GPs. Communication issues between health care providers and patients can also limit effective diabetes management and medication intensification. For instance, clinicians may have assume incorrectly that their patients are unable or reluctant to adapt to an increasingly complex regimen. For many patients, the fear of becoming dependent on insulin or a misunderstanding of the severity of the disease outweighs the physical fear of injections and injection discomfort that physicians perceive to be the most significant sources of concern [121]. This perception may lead to an inadequate education and understanding of the disease process and the importance of meeting the glycemic targets.

(c) system level: Several health-system-level issues can also lead to difficulty in achieving therapy goals. A lack of knowledge about guidelines or a lack of clear guidelines, differences among societies’ recommendations [122], and changing targets can contribute to the clinicians’ uncertainty about intensifying medication plans. This confusion is further complicated by the cost of drugs and changing formulary constraints, which are out of the control of patients and providers but can often influence care. For instance, a systematic review showed that patients with nonmedical switching—that is, the change in a patient’s prescribed medication to a different medication for reasons related to price, insurance coverage, formulary changes, and other administrative reasons [123]—used significantly fewer antihyperglycemic products compared with patients without nonmedical switching [124]. Moreover, time constraints placed on providers, as well as the lack of an institutional organization of care may further limit the health care system’s ability to provide consistent and effective care tailored to individual patients’ needs [125].

All these factors (i.e., patients, providers, and health-system-related factors) can contribute to therapeutic inertia and, in turn, have a large impact on outcomes for patients with DKD. Mechanisms to improve the communication between clinicians and patients as well as understanding the barriers to patients’ willingness or ability to engage in therapy are therefore essential to preventing therapeutic inertia, improving outcomes, and increasing medication-taking.

## 8. Strategies to Optimize the Management of DKD Patients

The management of DKD must be improved and is still far from perfect. The barriers for DKD treatment intensification must be crossed, and the common issues must be solved at all the three levels included in the intrinsic meaning of therapeutic inertia:

(A) Patient level: Diabetic patients should be conscious of the care plans and target value for the best DKD management: glucose, creatinine, GFR, blood urea nitrogen, phosphorus, calcium, PTH, Vitamin D, albumin, lipid, potassium, and hemoglobin targets. A proper management of blood pressure control and pulse pressure targets is essential. The patient should be motivated to follow a balanced dietary intake and know the best nutrients to choose to reach the desirable glucose values: the subject should use a pre-established meal plan and timing plan for glycemic control medication. Educational interventions should be performed for both the patient and family members, utilizing reading, virtual, and interactive educational materials.

(B) Clinician level: High-quality diabetes care requires creating a multi-specialist team that can gain a complete vision of the patient’s status and study the best strategies for implementing cures. Bridging fundamental approaches to care optimization for general practitioners, diabetologists, dieticians, nephrologists, and pharmacologists is critical. The team must perform a “treat to success” management approach rather than a “treat to failure” strategy [126]. Specialists and general practitioners should co-work to make the patient conscious of the importance of a proper glycemic and pressure control. An adequate doctor-patient communication should be promoted. The team must constantly ensure that the patient fully understands the therapeutic modifications and his health status variations. Psychological help should be guaranteed by professionals, especially to treat depression-related symptoms or to gradually overcome the denial of the disease.

A pharmacological consult should be considered in patients with complex multidrug therapy or in case of any possible doubt on the part of the clinician. Pharmacotherapy expertise can solve insidious drug-related problems in patients with a rapid decline of kidney function with many comorbidities [127]. A nephrological consult can be crucial in the process leading from DKD to a potential ESRD. A nephrological consult at the proper time can be a key point for delaying DKD complications and reducing ESRD risk factors. For DKD patients at high risk of ESRD, a transitional ambulatory can be a resolutive solution to improve the quality of life and reduce the risk of major adverse events and avoidable hospitalization. Physicians must avoid “educational inertia”, providing updated information on potential therapies and DKD outcomes to the patients [128]. The attention to modifiable risk factors can be decisive in reducing DKD progression risk factors. The medical team must promote smoking cessation counseling and regular physical activity. For this purpose, the educational interventions could be essential to the patient’s management.

An adequate educational training should also be performed for the clinicians, who must test their own performance and be aware of medical updates. Clinical audits must also be an integral part of the educational programs for health care professionals. Finally, cost-benefit data on drug use must be clearly explained and presented to the patient, who must freely evaluate and understand all the therapeutic strategies.

(C) System level: Specialized efforts to identify patients at high risk of DKD progression are of pivotal importance to program primary care strategies and to direct clinical resources. The health system must promote the necessary acts to improve the quality of care and establish clear guidelines among the different scientific societies to recognize subjects who may benefit from a closer control, intensive glucose-lowering treatment, or particular therapies. An implementation of data on therapeutic inertia should be performed globally: most of the studies were conducted in North America and in Europe, while in other Countries data are still scarce [129]. For this reason, DKD registries must be improved worldwide to monitor the standards of care and to establish the best strategies.

Real-world data use can also be helpful to assist physicians in making decisions about a patient’s care pathway.

A summary of these suggestions can be found in Table 3.

## 9. Future Perspectives for DKD-Related Therapeutical Inertia Management

Several studies are bringing evidence and suggestions to develop novel approaches to contrast therapeutic inertia in CKD and diabetic patients.

A review performed by Wrzal et al. [130] showed how in-person education is still the most relevant approach toward patients and between clinicians (54%), while technology still has a secondary role, with e-learning (14%), electronic medical records (8%), or mobile applications (18%). Interestingly, no educational intervention has focused on explaining the drugs’ side effects to the patients or reassuring them about the risk of hypoglycemia, rather showing a conservative approach of clinicians to therapeutic decisions. The main outcomes measured in the intervention group were improved glycated hemoglobin levels, a higher proportion of intensified therapies with insulin or GLP-1-RA, or better results in the knowledge tests provided [130].

As emerging from the observation by Reach et al. [131], the need for injectable medication corresponds to the time with the highest risk of therapeutic inertia in T2DM patients.

The need for new strategies to address this problem has been recently highlighted in several randomized and cluster-randomized controlled studies or prospective cohort trials, aiming to establish appropriate strategies directed to either the patient, clinician and/or, system levels, with only 28% of studies contemporarily addressing several levels [130].

ADA is currently conducting a 3-year initiative called Overcoming Therapeutic Inertia (OTI), to promote a better adherence to guideline indications in primary care settings. It is even developing user-friendly tools to support decision-making processes, in order to adopt therapeutic choices tailored to patients’ needs. Clinicians’ awareness of newer drugs and their positive effect on CV and renal outcomes, as well as a better knowledge of continuous glucose monitoring, represent a worldwide issue and are some of the practical steps to be addressed by the OTI initiative, as well as the identification of best practices with the constitution of a research team [132].

Health care system strategies should include the establishment of multidisciplinary care programs, as seen in other chronic diseases, such as CKD. These programs were shown to reduce medical costs and the need for RRT, slowing the progression to ESRD [133]. Diabetic patients should have access to a team composed of general practitioners, diabetologists, nephrologists, dieticians, and nurses to optimize self-care. Helou et al. [134] performed a crossover study on 32 participants (aged 67.8 ± 10.8), randomized into four arms, treated twice with three months of usual care alternated with three months of multidisciplinary management. The intervention improved general dietetic habits (55.43 vs. 38.31; *p* = 0.002), diabetes diet habits (56.84 vs. 37.02; *p* = 0.000), and blood sugar testing (53.84 vs. 39.77; *p* = 0.008; 95% CI), although the glycemic control and renal function indicators were similar for the intervention arms and the usual care.

Telemedicine may represent a new strategy to improve patients’ education and compliance with treatment. The Simultaneous Risk Factor Control Using Telehealth to slow the Progression of Diabetic Kidney Disease (STOP-DKD, NCT01829256) [135] focused on chronic disease management, including blood pressure self-monitoring and medication consult, provided by a clinical pharmacist for 36 months over the telephone. The trial did not reach the primary outcome of reducing the progression of CKD in the intervention arm vs. the usual care group but pointed out some challenges which should be taken into account in future studies, such as the need for a specific sub-population analysis based on demographic or clinical features, a less invasive role of healthcare professionals in monitoring patients, without overwhelming them with redundant or, worse, with contradictory communications [136].

## 10. Conclusions

DKD subjects present patient-related, clinician-related, and system-related issues. All these problems are translated into therapeutic inertia and a lower quality of care.

The health complexity of patients with many comorbidities makes a broader vision necessary, studying all their comorbidities. In turn, the patient should not be considered an isolated individual, but a component of a society that interacts with him. The clinician must consider not only the patient’s needs but also his limits. The clinician’s role is to find the best way to improve the quality of life of his patient, taking the time to communicate with him and to find the best strategies to reach the pharmacological targets appropriately. The third component of the therapeutic inertia, the “system”, must take into account the complexity of the interaction of the patient with his caregivers, medical specialists, and other figures that take care of his health.

Therapeutic inertia can be resolved by an evaluation of an interprofessional pool of health care experts. The timing of the intensification of treatment, the transition to the best therapy, and dietetic strategies must be provided by a multidisciplinary team, driving the patients to the glycemic target and delaying or overcoming DKD-related complications. A timely nephrologist evaluation can also guarantee adequate information to choose the right renal replacement therapy at the right time in case of renal impairment progression. The pharmacology consultation can facilitate the drug choices, to avoid prescribing inappropriate and potentially dangerous pharmacological interactions.

Future studies are necessary to improve the effective strategies to cross the therapeutic inertia barriers and to guarantee a patients-centered care with the best drug prescription at the right time.

## Figures and Tables

**Table 1:** Dose adjustment for antihyperglycemic drugs in DKD.

Drug Class	Medications	Recommendation
Biguanides	Metformin	Contraindicated if GFR <30 mL/min/1.73 m^2^ Not started in GFR 30–45 mL/min/1.73 m^2^
SGLT2 inhibitors	Empagliflozin	Avoid use or discontinue if GFR <45 mL/min/1.73 m^2^
	Canagliflozin	Avoid use if GFR <30 mL/min/1.73 m^2^ Dose adjustment in GFR 30–59 mL/min/1.73 m^2^
	Dapagliflozin	Contraindicated if GFR <30 mL/min/1.73 m^2^ Not started in GFR 30–45 mL/min/1.73 m^2^
First-generation sulfonylureas	Acetohexamide, tolazamide, tolbutamide, chlorpropamide	Avoid use
Second-generation sulfonylureas	Glyburide	Avoid use
	Glimepiride	Start cautiously in GFR <15 mL/min/1.73 m^2^
	Glipizide	No dose adjustment
	Glicazide	No dose adjustment
Alpha-glucosidase inhibitors	Acarbose	Contraindicated if GFR <30 mL/min/1.73 m^2^
GPL-1 receptor agonists	Exenatide	Contraindicated if GFR <30 mL/min/1.73 m^2^
	Lixisenatide	Contraindicated if GFR <15 mL/min/1.73 m^2^
	Liraglutide	No dose adjustment
	Albiglutide	No dose adjustment
	Dulaglutide	No dose adjustment
Thiazolidinediones	Pioglitazone	No dose adjustment
	Rosiglitazone	No dose adjustment
Meglitinides	Repaglinide	Start cautiously in GFR <15 mL/min/1.73 m^2^
DPP-4 inhibitors	Sitagliptin	Lower dosage
	Vildagliptin	Lower dosage
	Saxagliptin	Lower dosage
	Alogliptin	Lower dosage
	Linagliptin	No dose adjustment
Insulins	Dose adjustment based on patient response	

Abbreviations: DPP-4 = dipeptidyl peptidase-4; GFR = glomerular filtration rate; GPL-1 = glucagon-like peptide-1; SGLT2 = sodium-glucose cotransporter 2.

**Table 2:** Dose adjustment for statins in DKD.

Statins	Normal to Mildly Decreased (GFR: ≥90 to 60–89 mL/min/1.73 m^2^)	Mildly/Moderate Decreased to Kidney Failure (GFR: 45–59 to <15 mL/min/1.73 m^2^)
Lovastatin	No dose adjustment	NA
Fluvastatin	No dose adjustment	80 mg/day
Atorvastatin	No dose adjustment	20 mg/day
Rosuvastatin	No dose adjustment	10 mg/day
Simvastatin/Ezetmibe	No dose adjustment	20 mg/day
Pravastatin	No dose adjustment	40 mg/day
Simvastatin	No dose adjustment	40 mg/day
Pitavastatin	No dose adjustment	2 mg/day

Abbreviations: GFR = glomerular filtration rate; NA = not available.

**Table 3:** Suggested strategies to contrast therapeutic inertia.

Strategies to avoid therapeutic inertia	Educational interventions for both patient and care givers, with reading, virtual, and interactive materials. Promotion of proper management of blood pressure control and pulse pressure targets even with telemedicine consult. Promotion of smoking cessation and regular physical activity as modifiable risk DKD progression risk factors. Balanced dietary intake providing indications about the best nutrients to choose to reach the desirable glycemic target. Pre-established meal planning and timing plan for glycemic control medication.	Promotion of an adequate doctor-patient communication to assess the full comprehension of therapeutic modifications and a proper glycemic and pressure control, to avoid “educational inertia”. Explanation to the patient of the cost-benefit balance of therapies. Guarantee a “treat to success” management approach rather than a “treat to failure” strategy. Creation of a multidisciplinary team to guarantee a complete vision of the patients’ status for cure implementation. Pharmacological consult in patients with rapid decline of kidney function and many comorbidities undertaking complex multidrug therapy.	Program primary care strategies to identify patients at high risk of DKD progression and to direct clinical resources. Establish clear guidelines among the different scientific societies to recognize subjects who may benefit from a closer control, intensive glucose-lowering treatment, or particular therapies. Improve DKD registries worldwide to monitor the standards of care and to establish the best strategies. Implementation of real-world data use to assist physicians in making decisions about a patient’s care pathway.

## Data Availability

This study did not report any new data.

## Abbreviations

DKD	Diabetes kidney disease
DM	Diabetes mellitus
WHO	World Health Organization
GFR	glomerular filtration rate
ESRD	end-stage renal disease
CV	cardiovascular
UACR	urine albumin/creatinine ratio
NDA	National Diabetes Audit
T1DM	Type 1 diabetes mellitus
T2DM	Type 2 diabetes mellitus
IAF	Institute for Alternative Futures
PKC	protein kinase C
NF-κB	nuclear factor kappa-light-chain enhancer of activated B cells
IL	interleukin
TNF	tumor necrosis factor
AGEs	advanced glycation end-products species
RAS	renin-angiotensin system
ACEi	angiotensin-converting enzyme inhibitors
ARB	angiotensin II receptor blockers
RR	relative risk
CI	confidence interval
AKI	acute kidney injury
ASCVD	atherosclerotic cardiovascular disease
MRA	mineralocorticoid receptor antagonists
HR	hazard ratio
SGLT2i	Sodium-glucose co-transporter-2 inhibitors
GLP-1 RA	glucagon-like peptide-1 receptor agonists
DPP-4i	dipeptidyl peptidase-4 inhibitors
PPAR	peroxisome proliferator-activated receptors
LDL-c	low-density lipoprotein—cholesterol
ADRs	adverse drug reactions
GPs	general practitioners
